# Development and validation of a predictive nomogram for high-risk thyroid nodules: a retrospective analysis of sedentary time, insomnia, and elevated weight

**DOI:** 10.3389/fonc.2026.1698466

**Published:** 2026-04-01

**Authors:** Xiaolan Sun, Changmao Dai, Jiao Chen, Xiaohong Hu, Liangqing Wu, Yuanfeng Yu, Xueping Li

**Affiliations:** 1 Deyang Hospital Affiliated Hospital of Chengdu University of Traditional Chinese Medicine, Chengdu, Sichuan, China; 2 Hospital of Chengdu University of Traditional Chinese Medicine, Chengdu, Sichuan, China

**Keywords:** lifestyle, nomogram, risk factors, sedentary time, thyroid nodules

## Abstract

**Background:**

Thyroid nodules are widely regarded as one of the most prevalent endocrine disorders, and high-risk thyroid nodules are gradually gaining attention due to their potential malignancy. Early detection and active intervention are key to improving prognosis. Therefore, establishing a predictive model for assessing the risk of high-risk thyroid nodules is crucial for adjunctive diagnosis.

**Methods:**

The clinical data of patients with thyroid nodules admitted to the Hospital of Chengdu University of Traditional Chinese Medicine from October 2023 to June 2024 were retrospectively analyzed. According to the Thyroid Imaging Reporting and Data System classification, the patients were divided into a low-to-moderate risk group and a high-risk group. Multivariate logistic regression analysis was used to explore the influencing factors of high-risk thyroid nodules, and a nomogram was constructed. Internal validation was conducted using bootstrap resampling methods. The predictive performance of the model was evaluated by comparing the area under the receiver operating characteristic curve, the calibration curve, and the decision curve.

**Results:**

A total of 164 patients with thyroid nodules were included in this study, with an average age of 42.31 years. Among them, 101 patients (61.59%) were diagnosed as high-risk for thyroid nodules. Dietary diversity score (OR: 0.773, 95% CI = 0.639-0.934) and nodule diameter (OR: 0.909, 95% CI = 0.871-0.95) were protective factors for high-risk thyroid nodules, while sedentary time of more than two hours per day (OR: 2.8, 95% CI = 1.276-6.148), the Athens Insomnia Scale score (OR: 1.078, 95% CI = 1.01-1.15), and elevated weight (OR: 1.049, 95% CI = 1.008-1.09) were independent risk factors (all P<0.05).

**Conclusion:**

The nomogram model we constructed shows good predictive performance for high-risk thyroid nodules after internal validation, and may serve as a practical tool to guide the formulation of disease prevention strategies.

## 1 Introduction

Thyroid nodules (TNs), characterized as discrete lesions within the thyroid gland, represent one of the most prevalent clinical thyroid conditions (1). The prevalence in the general population is close to 25% (2), while the figure is 36.9% in China (3). Most often, these nodules occur in individuals with normal thyroid function and do not present with any clinical symptoms. Even in the absence of symptoms, malignancy cannot be ruled out (4). Approximately 5% of TNs are proven to be malignant (5). Therefore, the main clinical challenge for patients with TNs is the early identification of high-risk nodules to reduce the potential for progression and improve clinical prognosis.

In the current practice in China, the routine diagnostic approaches for thyroid diseases encompass palpation of the thyroid gland and serum testing of thyroid function markers (6). In addition, ultrasound examination based on the risk stratification standards of the American College of Radiology (ACR) is also a commonly used tool for assessment and diagnosis (7). However, ultrasound exams can only evaluate the current growth status of nodules and cannot anticipate their future progression. Therefore, in addition to the Thyroid Imaging Reporting and Data System (TI-RADS) characteristics, many clinical factors—including impaired fasting blood glucose, dyslipidemia (8), diastolic blood pressure (DBP) (9), as well as the size, shape, margin, and echogenicity of the nodules (10)—are considered predictive of the development of nodules or malignant tumors. The discovery of TNs may cause some anxiety and stress for patients. Overdiagnosis and overtreatment have emerged as major concerns in the management of thyroid nodule conditions. Therefore, this study focuses on investigating and analyzing lifestyle factors, aiming to identify important predictive factors for the occurrence of high-risk nodules in this patient group and to construct a predictive model.

## 2 Methods

### 2.1 Study subjects

Data were collected through a combination of questionnaires and face-to-face consultations from patients with TNs who visited the Endocrinology Department of the Hospital of Chengdu University of Traditional Chinese Medicine from October 2023 to June 2024.

Inclusion criteria: Patients aged 18 and older with TNs and complete clinical data.

Exclusion criteria: Patients with severe liver or kidney dysfunction.

### 2.2 Data collection

1. General conditions: age, gender, height, weight (WT), body mass index (BMI), habitual residence, educational level, and type of employment.
2. Lifestyle: history of smoking and drinking, dietary habits, sleep onset time, as well as women’s menstruation and childbirth conditions, etc.
3. Thyroid nodule assessment: laterality, number, diameter (DIA), TI-RADS classification, presence of cervical lymphadenopathy, pathological subtype, etc.
4. Comorbidities: hypertension and diabetes. Diagnostic criteria: Hypertension is defined as follows (11): when measuring blood pressure on three non-consecutive days without the use of antihypertensive medication, a systolic blood pressure (SBP) ≥ 140 mmHg and/or a DBP ≥ 90 mmHg. A patient with a prior history of hypertension who is currently on antihypertensive medication is still diagnosed with hypertension even if their blood pressure is below 140/90 mmHg. Diabetes is defined as follows (12): a clear prior history of diabetes with the use of hypoglycemic agents or insulin, or a fasting blood glucose level ≥ 7.0 mmol/L on two consecutive occasions.
5. The Athens Insomnia Scale (AIS) (13) is used to record whether participants experience insomnia and its severity (**Supplementary Table 1**).
6. Depression, Anxiety, and Stress Scale-21 (DASS-21) (14) is used to assess the levels of depression, anxiety, and stress in participants (**Supplementary Table 2**).
7. The Dietary Diversity Score (DDS) (15) is developed based on the Chinese Dietary Guidelines and uses a 24-hour dietary recall survey. Participants earning one point for daily or nearly daily consumption of grain, vegetables, and fruits, and an additional point for similar frequency in fungi, bean products, nuts, meat, eggs, fish, and dairy products, contribute to their DDS. Scoring is applicable when these food items are consumed “almost every day or at least once a week.” The total potential score for each participant is capped at ten points.

### 2.3 Data preprocessing

The data were preprocessed using Microsoft Excel 2019. The size of each thyroid nodule was defined as the maximum diameter measured by ultrasound examination. When dealing with multiple nodules, where the count exceeds one, the nodule of the largest diameter was chosen for analysis.

### 2.4 Diagnostic criteria

Refer to the TI-RADS classification standards published by ACR in 2017 (7): (1) TR1 indicates a benign nodule; (2) TR2 indicates a malignancy possibility of ≤2%; (3) TR3 indicates a malignancy possibility of ≤5%; (4) TR4 is suspicious for a malignant nodule, with a malignancy possibility of 5% to 20%; (5) TR5 indicates a malignancy possibility of >20%.

### 2.5 Statistical analysis

SPSS Statistics Version 25.0 (IBM) was used for statistical analysis. Normally distributed data were presented as mean ± SD, with comparisons between the two groups conducted using the t-test. Non-normally distributed data were expressed as median (interquartile range), and the Mann–Whitney U test was employed for group comparisons. Categorical data were reported as frequency (percentage), and group comparisons were performed using the chi-square test.

A multivariate logistic backward regression analysis was performed to identify risk factors, with results expressed as odds ratios (OR) and 95% confidence intervals (CI). A p-value of less than 0.05 was deemed statistically significant. Based on the independent predictors identified from this analysis, a predictive nomogram was formulated *ad hoc* by the authors. Specifically, insomnia severity was quantified using the Athens Insomnia Scale, while the other variables (including elevated weight, sedentary time, dietary diversity score, and nodule diameter) were collected via our questionnaire. The nomogram thus represents an original model derived from our clinical data. The R 4.4.1 software package was used to construct a nomogram model for predicting high-risk TNs. Internal validation was performed using bootstrap resampling with 1,000 repetitions. The receiver operating characteristic (ROC) curve was used to analyze the predictive value of the nomogram model. A calibration curve was used to assess the model’s adequacy in fitting the data, while a decision curve was constructed to evaluate the clinical efficacy of the nomogram model.

## 3 Results

### 3.1 General characteristics of the study population

**Figure 1:**
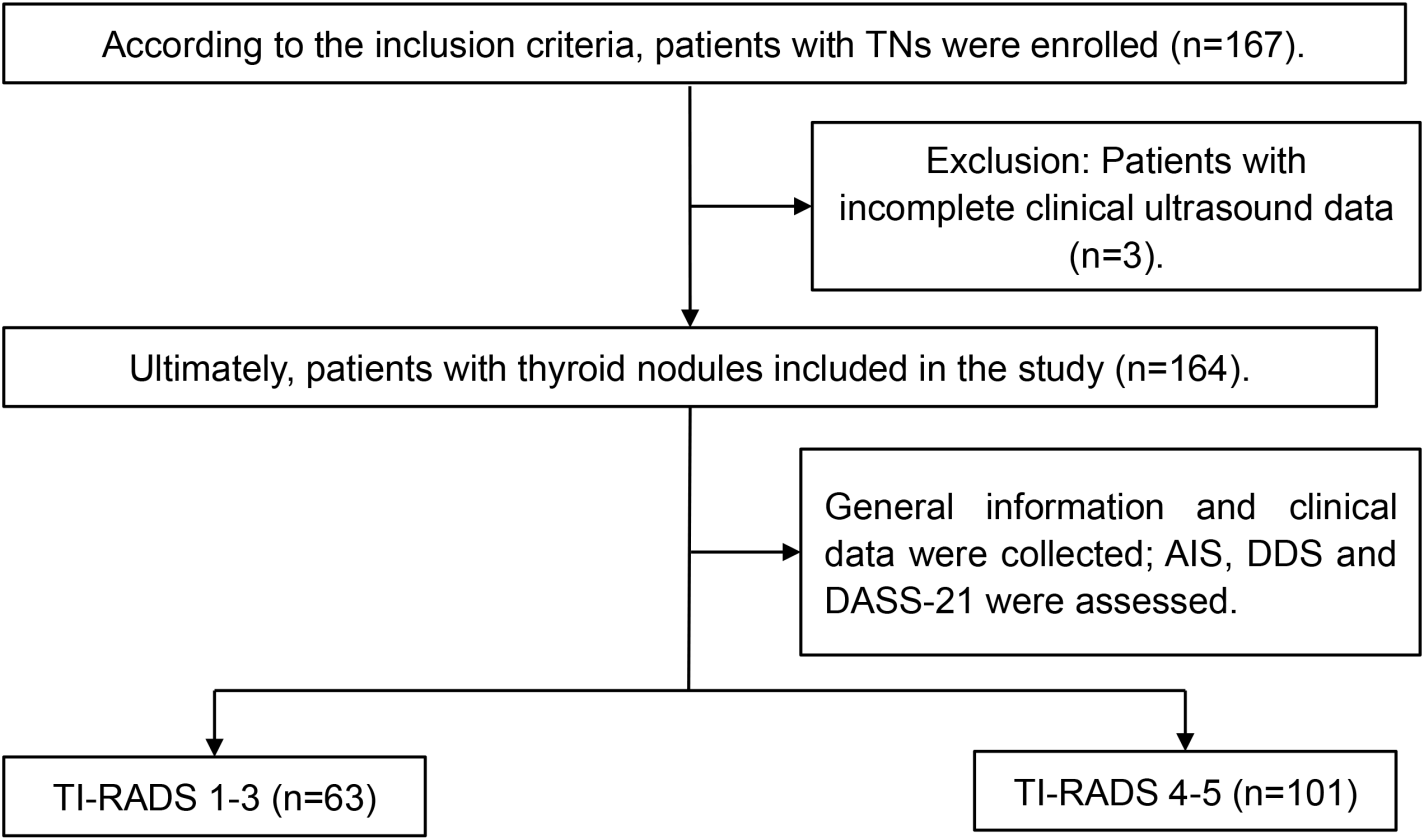
Flow diagram illustrating the selection of study participants.

According to the inclusion and exclusion criteria, a total of 164 patients with TNs were included in this study, with a male-to-female ratio of approximately 2:8 (38:126). The ages ranged from 18 to 73 years, with an average age of 42.31 ± 11.28 years. There were 63 cases classified as TI-RADS 1-3 (including 5 cases in TI-RADS 1–2 and 58 cases in TI-RADS 3), accounting for 38.41% of the total; 101 cases were classified as TI-RADS 4-5, accounting for 61.59% (**Figure 1**). Among the 164 patients with TNs, 7 had diabetes, 14 had hypertension, and 2 had both conditions. Additionally, 27 patients had a family history of thyroid disease.

### 3.2 Comparison of the general conditions between the low-to-moderate risk group and the high-risk group

**Figure 2:**
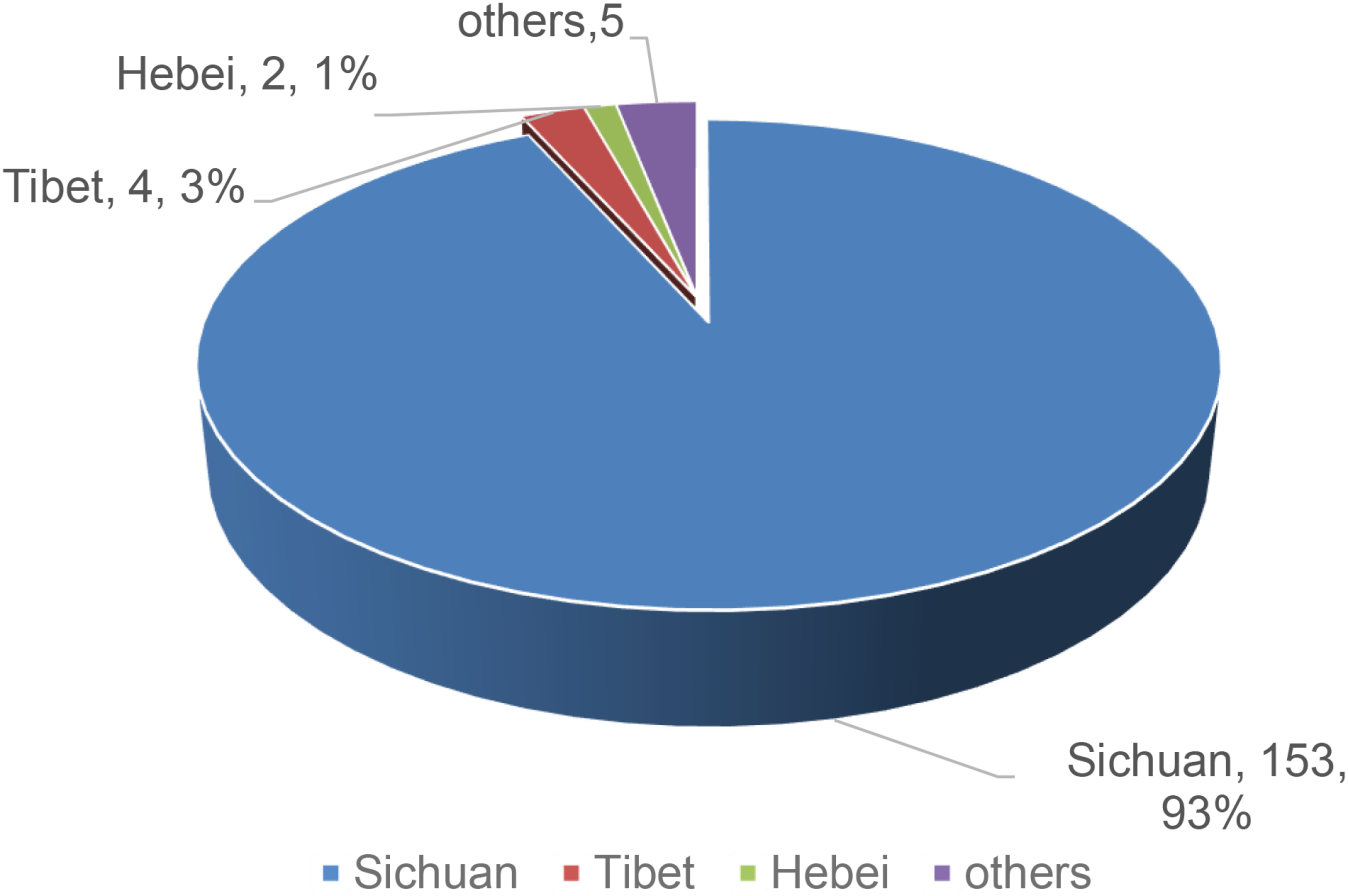
Distribution of participants’ habitual residences.

This study categorized TNs into three groups based on TI-RADS classification: low, moderate, and high risk. TI-RADS 1, 2, and 3 were collectively labeled as “ low-to-moderate risk,” while TI-RADS 4 and 5 were categorized as “high risk”. Univariate analysis was performed on 28 research factors, including gender, age, smoking and drinking history, sleep, and depression/anxiety/stress scales, etc. The results were shown in **Table 1**. Both groups had a similar proportion of patients with a college education or above and of those engaged in intellectual labor, with a similar distribution. The high-risk group had a lower average age (M = 39 years) compared to the low-to-moderate risk group (M = 45 years). The BMI in the high-risk group (24.05 ± 3.40) was higher, and the count of patients exposed to radioactive materials more than once a year (83 cases) was greater, constituting a higher proportion (82.18%). In terms of lifestyle, the overall drinking rate in the high-risk group (39.60%) was higher, with a variety of drinking habits, and the proportion of late sleepers (65.35%) was also higher. As shown in **Figure 2**, 93% of participants were from Sichuan, where spicy food is predominant; thus, both groups showed the highest proportion of spicy food consumers (all P > 0.05).

**Table 1:** Comparison of the general conditions between the low-to-moderate risk group and the high-risk group.

Variables	Low-to-moderate risk group (n=63)	High-risk group (n=101)	Z/t/χ^2^	P value
Female	49(77.78%)	77(76.24%)	0.052	0.820
Age, years	45(35,51)	39(33,49.5)	-1.367	0.172
Height, m	1.60(1.56,1.65)	1.62(1.56,1.65)	-1.000	0.317
Weight, kg	59(53,66)	63(55,70)	-2.063	0.039
BMI, kg·m^-2^	23.02±3.12	24.05±3.40	-1.945	0.053
College education or above	26(41.27%)	48(47.52%)	-0.708	0.479
Smoking	15(23.81%)	20(19.80%)	0.371	0.542
Drinking	17(26.98%)	40(39.60%)	2.725	0.099
Drinking Baijiu	11(17.46%)	21(20.79%)	0.274	0.601
Drinking red wine	5(7.94%)	20(19.80%)	4.228	0.040
Drinking beer	12(19.05%)	26(25.74%)	0.977	0.323
Exposure to radioactive substances more than once a year	45(71.43%)	83(82.18%)	2.617	0.106
Mental labor work	29(46.03%)	47(46.53%)	0.004	0.950
ST > 2 hours per day	36(57.14%)	75(74.26%)	5.196	0.023
Working on a shift schedule	2(3.17%)	8(7.92%)	0.810	0.368
Falling asleep after 11 PM	35(55.56%)	66(65.35%)	-1.597	0.110
Sleep duration of 6 hours or less per day	15(23.81%)	19(18.81%)	-0.580	0.562
DDS	6(4,8)	5(4,6)	-3.181	0.001
Preferring seafood	15(23.81%)	13(12.87%)	3.279	0.070
Preferring spicy food	36(57.14%)	69(68.32%)	2.103	0.147
Preferring raw and cold food	9(14.29%)	12(11.88%)	0.201	0.654
Preferring sweets	24(38.10%)	44(43.56%)	0.478	0.489
Eating after 10 PM more than 3 times a week	2(3.17%)	5(4.95%)	-1.044	0.296
Eating spicy foods more than once a week	9(14.29%)	14(13.86%)	-1.938	0.053
AIS	15(9,19)	17(11,21)	-2.057	0.040
Stress Scale	22(16,28)	24(18,30)	-1.940	0.052
Anxiety Scale	18(16,24)	20(16,18)	-2.441	0.015
Depression Scale	18(14,22)	20(16,26)	-2.441	0.016

BMI, body mass index; ST, sedentary time; PM, post meridiem; DDS, dietary diversity score; AIS, Athens Insomnia Scale.

Patients in the high-risk group had a higher weight (M = 63 kg) compared to the low-to-moderate risk group (M = 59 kg). Additionally, more patients consumed red wine and spent over 2 hours sitting each day. In the high-risk cohort, the DDS was observed to be lower compared to those in the low-to-moderate risk cohort. Conversely, scores on the AIS, anxiety scale, and depression scale were notably higher in the high-risk group. The analysis revealed that these seven factors had a significant impact on the high risk of TNs (P < 0.05).

### 3.3 Comparison of clinical data between the low-to-moderate risk group and the high-risk group

Clinical data from the two groups were compared by gathering patients’ personal and family histories, along with relevant ultrasound data. As shown in **Table 2**, the diameter of nodules in the high-risk group (M = 8 mm) was significantly smaller than that in the low-to-moderate risk group (M = 18 mm). Furthermore, no statistically significant differences were observed between the two groups concerning nodule laterality, count, and the presence or absence of enlarged cervical lymph nodes (all P > 0.05).

**Table 2:** Comparison of clinical data between the low-to-moderate risk group and the high-risk group.

Variables	Low-to-moderate risk group (n=63)	High-risk group (n=101)	Z/t/χ8^2^	P value
Laterality (bilateral)	33(52.38%)	48(47.52%)	0.366	0.545
Number (multiple)	40(63.49%)	60(59.41%)	0.272	0.602
Diameter, mm	18(8,30)	8(5,14)	-4.697	<0.001
Enlarged cervical lymph nodes	17(26.98%)	25(24.75%)	0.101	0.750
Hypertension	6(9.52%)	8(7.92%)	0.128	0.721
Diabetes	2(3.17%)	5(4.95%)	0.299	0.584
Family history of thyroid disease	12(19.05%)	15(14.85%)	0.497	0.481

### 3.4 Comparison of clinical data between the cancer group and the non-cancer group

Among the 101 patients with TNs classified as TI-RADS 4-5, a total of 79 patients underwent needle biopsy for pathological examination. The biopsy results revealed that 37 patients were diagnosed with thyroid cancer, resulting in a detection rate of 53.16%. Of these, there were 36 cases of papillary carcinoma, accounting for 85.71%; 2 cases of follicular carcinoma, accounting for 4.76%; and 4 cases of anaplastic carcinoma, accounting for 9.52%. Based on the pathological biopsy results, the 79 patients were categorized into a cancer group and a non-cancer group for clinical data comparison, as shown in **Table 3**, with no significant differences observed (P > 0.05).

**Table 3:** Comparison of clinical data between the cancer group and the non-cancer group.

Variables	Cancer group (n=37)	Non-cancer group (n=42)	Z/t/χ^2^	P value
Laterality (bilateral)	24(64.86%)	18(42.86%)	0.908	0.341
Number (multiple)	22(59.46%)	20(47.62%)	0.110	0.741
Diameter, mm	8(6.25,16.5)	8.5(5,12)	-0.694	0.488
Enlarged cervical lymph nodes	27(72.97%)	15(35.71%)	3.832	0.05
Hypertension	1(2.70%)	2(4.76%)	–	1.000
Diabetes	1(2.70%)	1(2.38%)	–	1.000
Family history of thyroid disease	5(13.51%)	6(14.29%)	0.010	0.921

### 3.5 Comparison of menstrual and childbirth histories among female patients

As shown in **Table 1**, both groups had a higher number of female patients, with significant proportions: 49 cases (77.78%) in the low-to-moderate risk group and 77 cases (76.24%) in the high-risk group. This may be attributed to elevated estrogen levels (16). Therefore, univariate analysis was conducted on the menstrual, childbirth history, and other conditions of female patients (**Table 4**). The differences in age at menarche, menopausal status, childbirth, miscarriage, and the presence of breast nodules between the two groups of female patients were not statistically significant (P > 0.05).

**Table 4:** Comparison of menstrual and childbirth histories among female patients.

Variables	Low-to-moderate risk group (n=49)	High-risk group (n=77)	Z/t/χ^2^	P value
First menstruation after age 13	35(71.43%)	47(61.04%)	1.422	0.233
Menopause	16(32.65%)	25(32.47%)	0.0005	0.983
Birth of one child	21(42.86%)	33(42.86%)	1.151	0.842
Miscarriage of one child	7(14.29%)	12(15.58%)	8.320	0.094
Breast nodule	17(34.69%)	23(29.87%)	0.399	0.809

### 3.6 Multivariate logistic regression analysis

**Figure 3:**
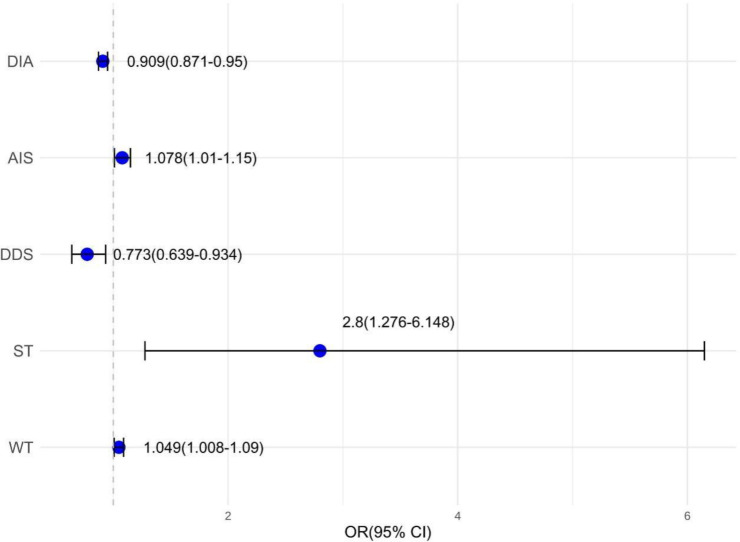
Multivariate Logistic Regression Analysis of High-Risk TNs. DIA, diameter; AIS, Athens Insomnia Scale; DDS, dietary diversity score; ST, sedentary time; WT, weight.

Using the high-risk status of TNs as the outcome variable, a collinearity diagnosis was performed on the eight statistically significant variables from **Table 3** and **4**, including weight, sedentary time > 2 hours per day, nodule diameter, etc. Since all variance inflation factor (VIF) values were < 5, it was considered that there was no collinearity among the variables. These eight univariate factors were incorporated into the logistic regression model, which utilized a stepwise backward elimination method for the regression process. Ultimately, five variables were included in the model (**Figure 3**), based on an inclusion criterion of P < 0.05. The Hosmer-Lemeshow test for this regression model yielded a p-value greater than 0.05, indicating a good fit.

It is worth noting that while red wine consumption, anxiety scale scores, and depression scale scores showed statistical significance in univariate analysis, they were not retained in the final multivariate model. Several methodological factors may explain this observation. First, the relatively modest sample size (n = 164) may have provided insufficient statistical power to detect independent effects of psychological factors after accounting for stronger lifestyle predictors. Second, although collinearity diagnostics (VIF < 5) indicated no severe multicollinearity, the anxiety and depression subscales of DASS-21 may capture symptom domains that overlap conceptually with insomnia (assessed by AIS), potentially contributing to their attenuation in the multivariate context. Third, as this is a single-center study, the possibility that these findings reflect characteristics specific to our study population cannot be excluded; external validation in larger, diverse cohorts is necessary to determine whether this variable selection pattern is replicable.

### 3.7 Development and evaluation of the predictive model

#### 3.7.1 Development of the predictive model

**Figure 4:**
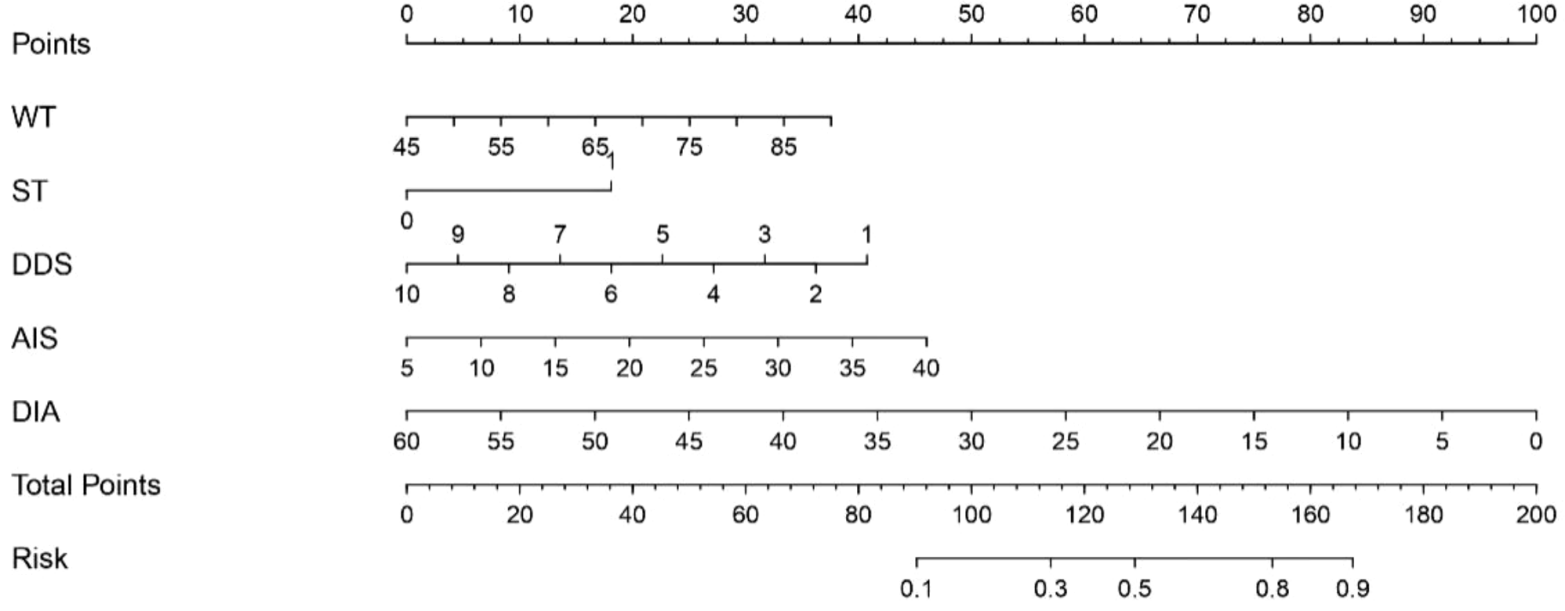
Prediction nomogram model for high-risk TNs. WT, weight; ST, sedentary time; DDS, dietary diversity score; AIS, Athens Insomnia Scale; DIA, diameter.

We found that red wine consumption, scores on the anxiety scale, and the depression scale were not independent risk factors for high-risk status in patients with TNs (P > 0.05). However, the DDS (OR = 0.773, 95% CI: 0.639–0.934, P < 0.05) and nodule diameter (OR = 0.909, 95% CI: 0.871–0.950, P < 0.05) were recognized as protective factors, while elevated weight (OR = 1.049, 95% CI: 1.008–1.090, P < 0.05), sedentary time exceeding two hours per day (OR = 2.800, 95% CI: 1.276–6.148, P < 0.05), and higher AIS scores (indicating more severe insomnia; OR = 1.078, 95% CI: 1.010–1.150, P < 0.05) were identified as independent risk factors. Among these, sedentary time demonstrated the strongest association with high-risk thyroid nodules, with an odds ratio of 2.800, indicating that patients with prolonged sedentary behavior had nearly three times the odds of presenting with high-risk nodules. These five statistically significant variables, derived from multivariate logistic regression analysis, were used to develop a nomogram model formulated *ad hoc* by the authors for predicting the high-risk condition of thyroid nodules (**Figure 4**).

Several methodological factors may explain the observed protective effect of smaller nodule diameter. First, selection bias cannot be ruled out: patients with larger nodules may have been more likely to undergo immediate biopsy or surgery rather than being included in this observational study, potentially skewing the sample toward smaller nodules with higher-risk features. Second, measurement artifacts related to ultrasound assessment may have influenced the results. It should be emphasized that this finding does not suggest that smaller nodules should be prioritized for intervention, but rather highlights the complexity of thyroid nodule risk assessment and the importance of dynamic monitoring. Prospective multi-center studies with standardized ultrasound protocols are urgently needed to clarify the relationship between nodule size and malignancy risk across diverse populations and clinical settings.

#### 3.7.2 Evaluation of the predictive model

**Figure 5:**
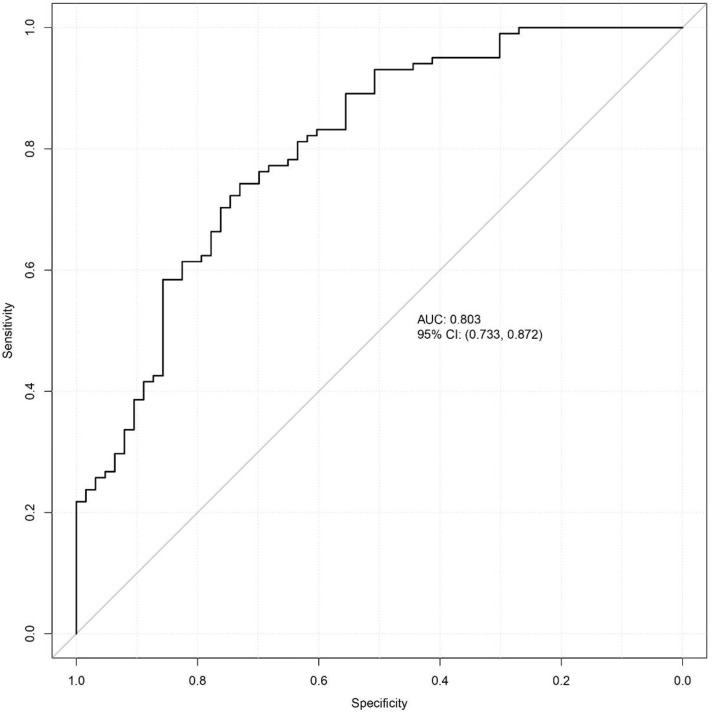
ROC curve for the nomogram model predicting high-risk TNs. AUC, area under the curve; CI, confidence intervals.

**Figure 6:**
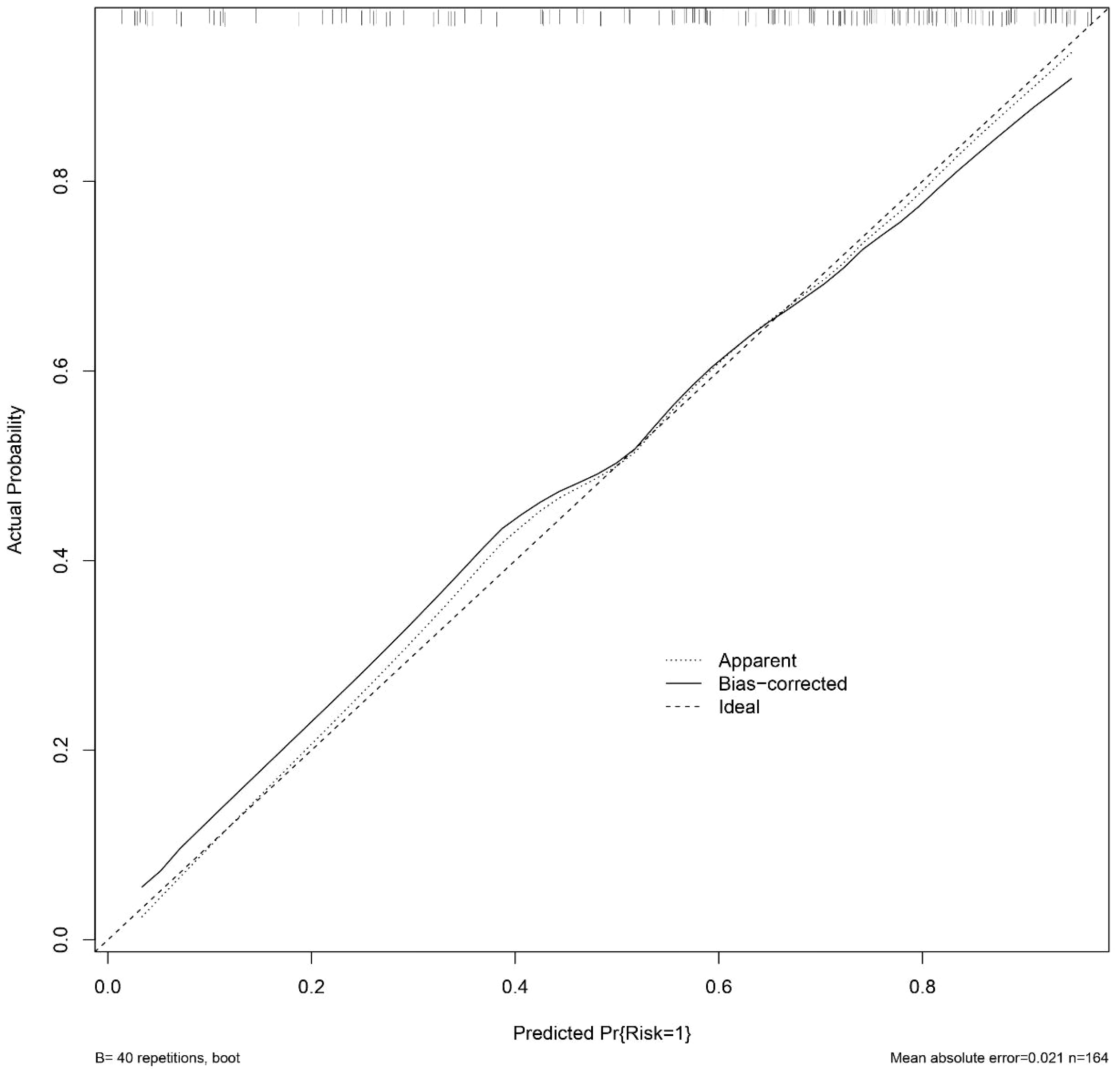
Calibration curve for the nomogram model predicting high-risk TNs.

**Figure 7:**
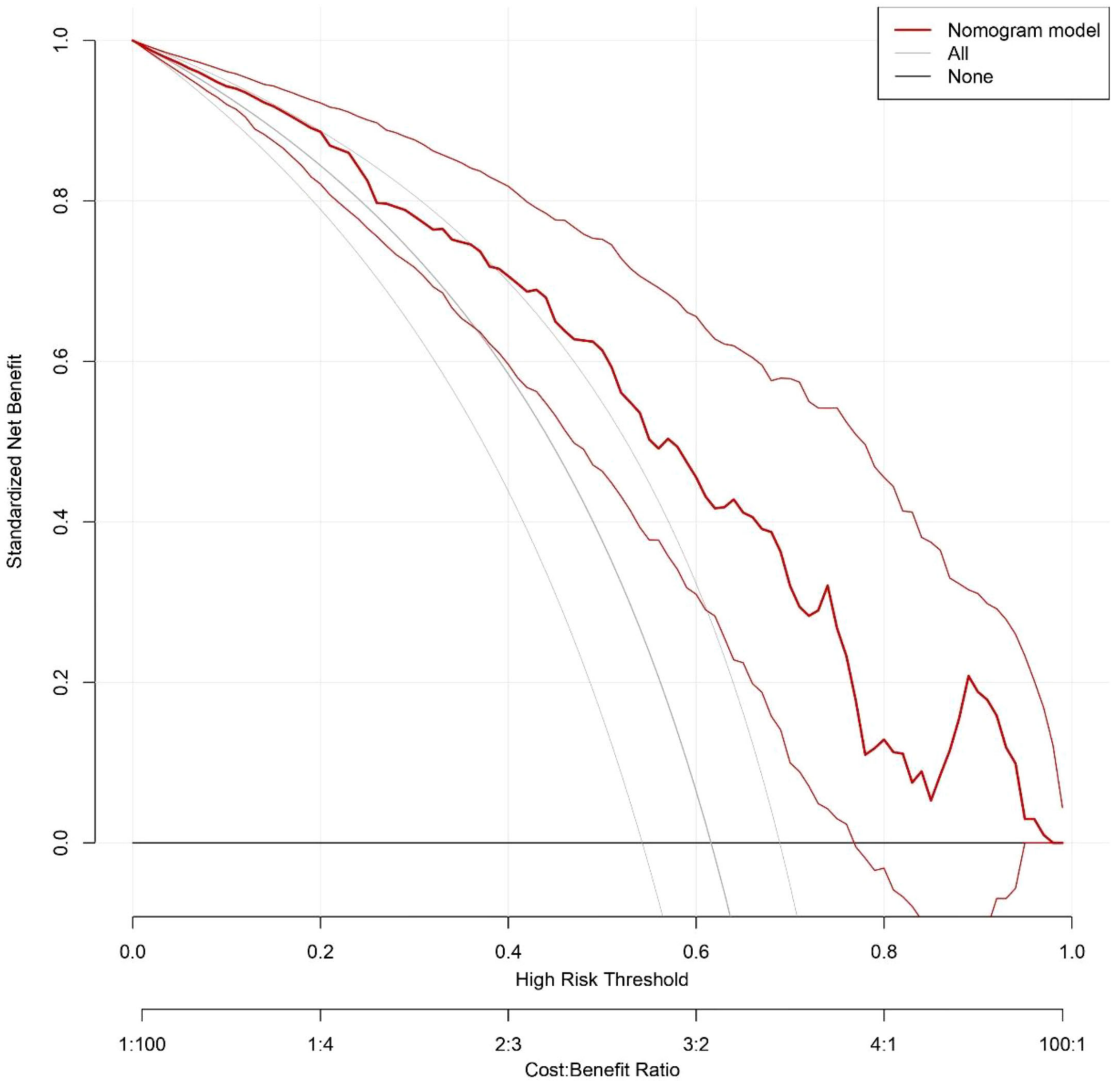
Decision curve for the nomogram model predicting high-risk TNs.

Bootstrap resampling was performed 1,000 times, and the results indicated that the consistency index (C-index) of the nomogram model was 0.803. The analysis of the ROC curve (**Figure 5**) revealed that the area under the curve (AUC) for the nomogram model aimed at predicting high-risk TNs was 0.803, with a 95% confidence interval spanning from 0.733 to 0.872. Both the apparent line and the bias-corrected line of the calibration curve showed slight fluctuations near the ideal line. The model’s predictions, characterized by a mean absolute error of 0.021, generally aligned with the observed variables in the dataset, indicating robust predictive capacity (**Figure 6**). The decision curve analysis underscored that the red curve outperformed both the “none” and “all” lines, affirming that the model delivered superior net benefits across various high-risk thresholds (**Figure 7**).

## 4 Discussion

TNs have become one of the most common health issues globally (17). Approximately 5-10% of thyroid nodules carry a risk of malignant transformation (18), posing a threat to patients’ health and potentially causing anxiety. Therefore, individuals diagnosed with TNs must maintain regular check-ups and closely monitor the health status of their nodules. On the other hand, they should receive health education and adjust unhealthy lifestyles, such as irregular meal times, smoking, heavy physical labor, central obesity, anxiety, and depression (19, 20). Due to the stealthy onset of TNs, it presents an opportunity for related research. Therefore, identifying potential risk factors associated with the development of thyroid nodules is essential. Early detection and intervention can reduce or delay the occurrence of thyroid malignancies. This is a challenging but significant task.

The univariate analysis of this study indicated that, compared to the low-to-moderate risk group, the high-risk group exhibited significantly increased levels of sedentary time, frequency of red wine consumption, scores on the AIS, depression scale, and anxiety scale, and body weight. However, the levels of the nodule diameter and DDS were relatively lower. Using the results from the univariate variable selection, a multifactorial logistic regression model was constructed, identifying five independent diagnostic indicators. Among them, the DDS and nodule diameter were protective factors for high-risk TNs, while sedentary time greater than 2 hours per day, the AIS, and elevated weight were independent risk factors.

Iodine deficiency or sufficiency is recognized as an independent risk factor for thyroid disease, while dietary diversity reflects the adequacy of micronutrients in one’s diet (21). In areas with monotonous grain-based diets, most individuals experience thyroid diseases and micronutrient deficiencies, including vitamin A and iron deficiencies (22). A cross-sectional study in Ethiopia also found that children with low DDS were more likely to develop thyroid diseases (23). Therefore, a diversified diet not only provides the various nutrients required by the body but also ensures that we intake sufficient trace elements and vitamins necessary for maintaining normal thyroid function. This is consistent with our research findings, indicating that a balanced dietary structure, when combined with a diverse array of food sources, effectively reduces the incidence of high-risk TNs.

On the other hand, an analysis based on the Chinese TI-RADS showed that (24), regardless of benign or malignant status, the incidence of four positive features decreased with increasing nodule size. Another cross-sectional study (25) indicated that nodules smaller than 4 cm were more likely to show extrathyroidal extension, lymphovascular invasion, and positive surgical margins compared to those 4 cm or larger, although these differences were not statistically significant. Of course, research by Allison Cavallo (26) suggested that the size of a thyroid nodule is inversely related to the risk of malignancy. This remains a contentious topic, with inconsistent results and contradictory conclusions. In a clinical setting, it is essential to consider not only the nodule diameter but also other ultrasound characteristics and thyroid function tests to comprehensively assess the risk category of the nodule. Careful and dynamic monitoring of the risk changes associated with the nodule is necessary to minimize the risk of overdiagnosis and overtreatment. Consequently, comprehensive prospective studies are necessary to validate these findings.

Overall, the importance of lifestyle factors in managing thyroid nodules is receiving increasing attention. The results of this study indicated that sedentary time (>2 hours per day), insomnia, and elevated weight were three factors that increased the risk of TNs. While our sample size is modest, these three identified lifestyle factors have biological plausibility supported by existing literature. To our knowledge, this study is the first to observe the association between sedentary time and an increased risk of TNs. Sedentary behavior is common in both work and home environments. It’s associated with various adverse health conditions, such as chronic obstructive pulmonary disease (27), heart failure (28), and frailty (29). A comprehensive analysis of a large prospective study based in the UK revealed that sedentary time was significantly related to a higher risk of twelve non-communicable diseases (NCDs), including thyroid disease. Substituting sedentary time with a comparable amount of physical activity may decrease the risk of specific diseases (30).

The hypothalamic-pituitary-thyroid axis has been recognized as one of the neuroendocrine systems linked to the stress response (20). The presence of negative emotions such as stress, anxiety, and depression may play a role in the development of thyroid nodules (TNs). In this study conducted among patients with TNs, the related indicators of the DASS-21 did not emerge as final predictive indicators; instead, poor sleep quality stood out. Insomnia is prevalent among individuals with thyroid nodules and is also associated with an increased prevalence of differentiated thyroid cancer (31, 32). Therefore, within the limited 24-hour day, we recommend that patients focus on “reducing sedentary time, increasing physical activity, and ensuring good sleep” for better thyroid health. It is advisable to incorporate the assessment of sleep quality and guidance on physical exercise into the management of thyroid disease, using a multidisciplinary approach to develop individualized intervention strategies.

A cross-sectional study (33) from China indicated that obesity is a contributing factor for thyroid disorders, especially among individuals with thyroid nodules. Furthermore, a retrospective cohort study also suggested that being overweight significantly increases the cancer risk in patients with Graves’ disease (34). Elevated weight was recognized as a relevant risk factor for both TNs and thyroid cancer, with potential mechanisms linked to insulin resistance, chronic inflammatory states, and the production of leptin from adipose tissue (35). Therefore, as one of the most flexible lifestyle factors, controlling weight is an effective approach for quickly improving metabolic status and achieving clinical benefits.

Predictive models have become essential tools in various medical fields, including cardiovascular disease (36) and diabetes (37). These models enable rapid and accurate disease diagnosis, support individualized risk assessment, and provide critical information for clinical decision-making. TNs are widely regarded as one of the most common endocrine diseases. Due to the risk of malignancy, high-risk TNs are increasingly emphasized. Early detection and proactive intervention are key to improving prognosis. Therefore, the establishment of predictive models for high-risk TNs is essential for adjunctive diagnosis. This study showed that our prognostic nomogram model demonstrated strong discrimination and calibration, as evidenced by the ROC and calibration curves. It may serve as a valuable tool for identifying high-risk patients with TNs. Our findings align with contemporary perspectives that highlight the multifactorial nature of thyroid nodule risk and the growing role of artificial intelligence in enhancing diagnostic accuracy (38, 39). For clinical implementation, clinicians may use the nomogram to stratify patients into different risk categories based on their predicted probabilities, with higher values indicating greater likelihood of high-risk nodules warranting more aggressive management. However, optimal threshold selection requires further study and prospective validation.

## 5 Limitations

This study has several limitations. First, the nomogram was developed using single-center data from Sichuan province, China, with 93% of participants from this region. This geographic homogeneity, combined with the retrospective design and modest sample size (n = 164; 38.41% low-to-moderate risk), introduces potential selection bias and limits generalizability. The limited sample size may have reduced statistical power, potentially explaining the failure to detect associations with established risk factors such as prior radiation exposure or family history of thyroid disease. Consequently, some identified predictors—though statistically significant—could be population-specific artifacts rather than generalizable risk factors. To mitigate this risk, we performed bootstrap resampling (1,000 repetitions) for internal validation, which demonstrated model stability. However, internal validation cannot replace external validation. Therefore, while our nomogram shows promising predictive performance in this cohort, external validation using independent, multi-center cohorts from diverse geographic regions is essential before clinical application. Such studies should also assess model performance across populations with varying dietary patterns and lifestyle factors.

## 6 Conclusion

This study developed a nomogram for predicting high-risk thyroid nodules based on five variables: DDS, nodule diameter, sedentary time >2 hours/day, AIS score, and elevated weight. The model demonstrated robust predictive performance (AUC = 0.803) and clinical relevance. Our findings identify sedentary time, insomnia severity (assessed by AIS), and elevated weight as three key modifiable risk factors, with sedentary time showing the strongest association. These results highlight the importance of lifestyle factors in risk assessment, particularly prolonged sedentary behavior as a high-impact intervention target. The nomogram may assist clinicians in screening and treatment decisions.

## Data Availability

The raw data supporting the conclusions of this article will be made available by the authors, without undue reservation.
