# Supplementary material for: Development and validation of a predictive nomogram for high-risk thyroid nodules: a retrospective analysis of sedentary time, insomnia, and elevated weight
Source: Front Oncol. 2026 Apr 1;16:1698466. doi: 10.3389/fonc.2026.1698466 (PMC13080605; doi:10.3389/fonc.2026.1698466)
Supplement: Supplementary file 1 [file DataSheet1.pdf]

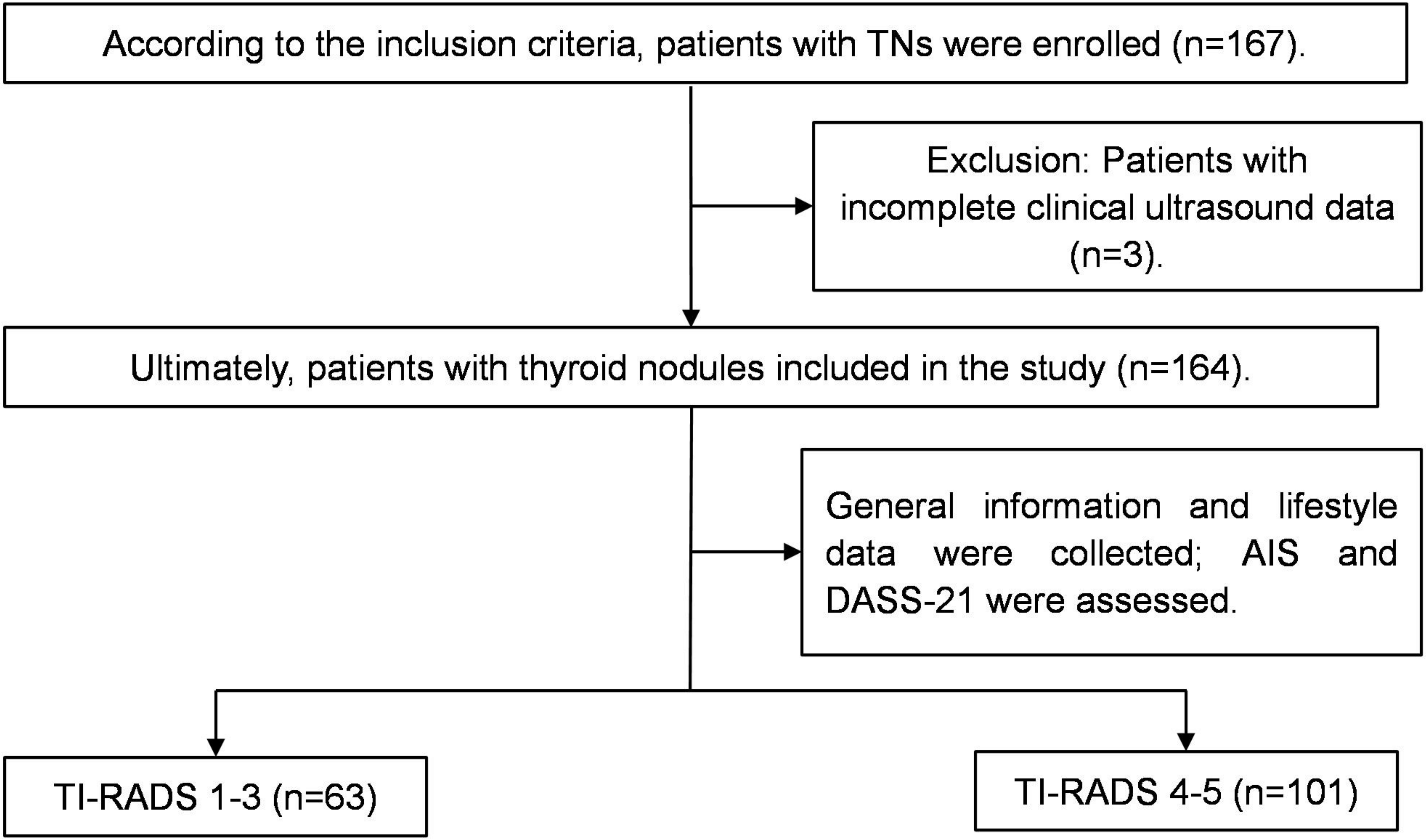
According to the inclusion criteria, patients with TNs were enrolled (n=167).
Exclusion: Patients with incomplete clinical ultrasound data (n=3).
Ultimately, patients with thyroid nodules included in the study (n=164).
General information and lifestyle data were collected; AIS and DASS-21 were assessed.
TI-RADS 1-3 (n=63)
TI-RADS 4-5 (n=101)
