# Supplementary figures and images for: Development and validation of a predictive nomogram for high-risk thyroid nodules: a retrospective analysis of sedentary time, insomnia, and elevated weight
Source: Front Oncol. 2026 Apr 1;16:1698466. doi: 10.3389/fonc.2026.1698466 (PMC13080605; doi:10.3389/fonc.2026.1698466)

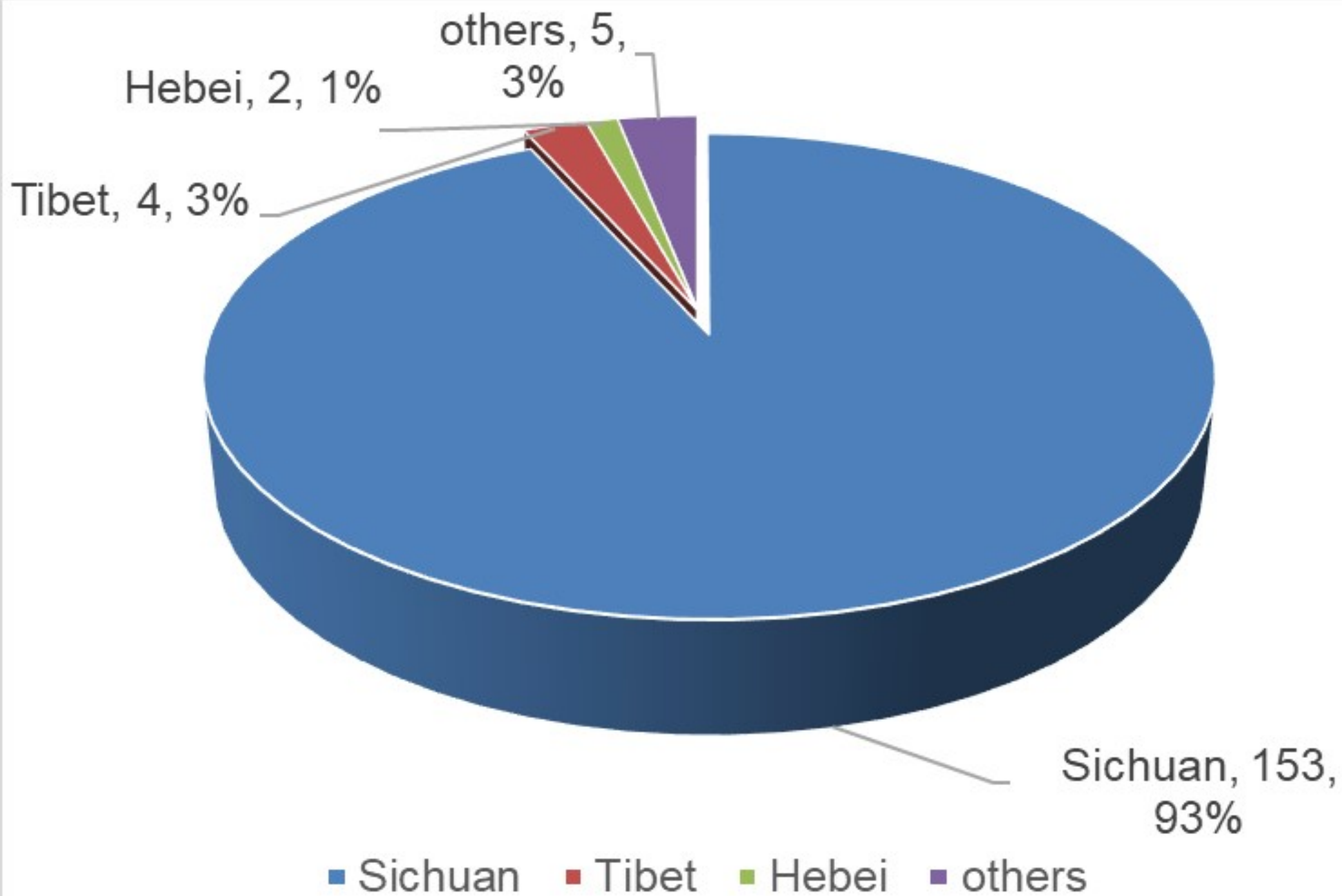

Supplement: Supplementary file 2 [file DataSheet2.pdf]

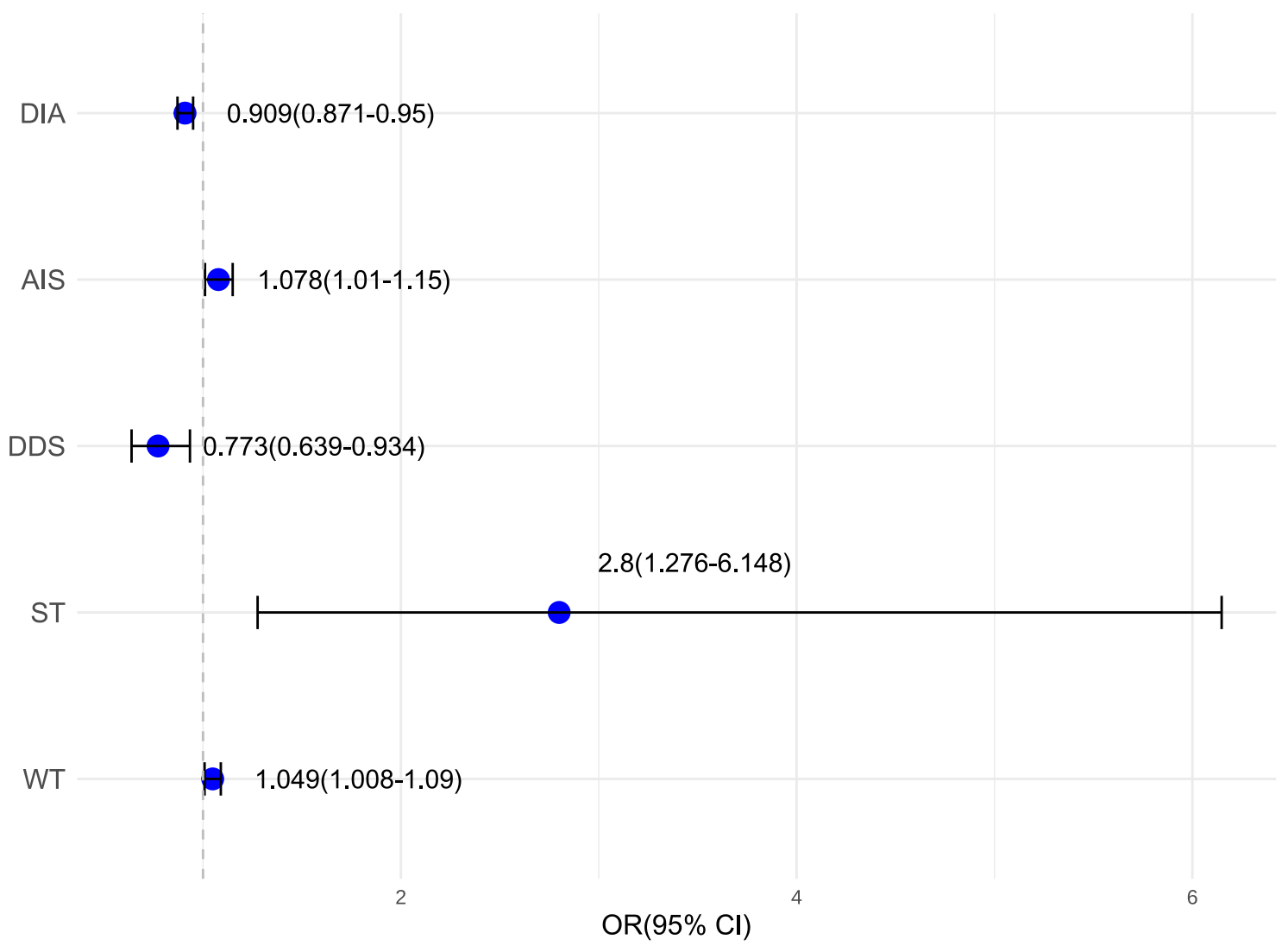

Supplement: Supplementary file 3 [file DataSheet3.pdf]

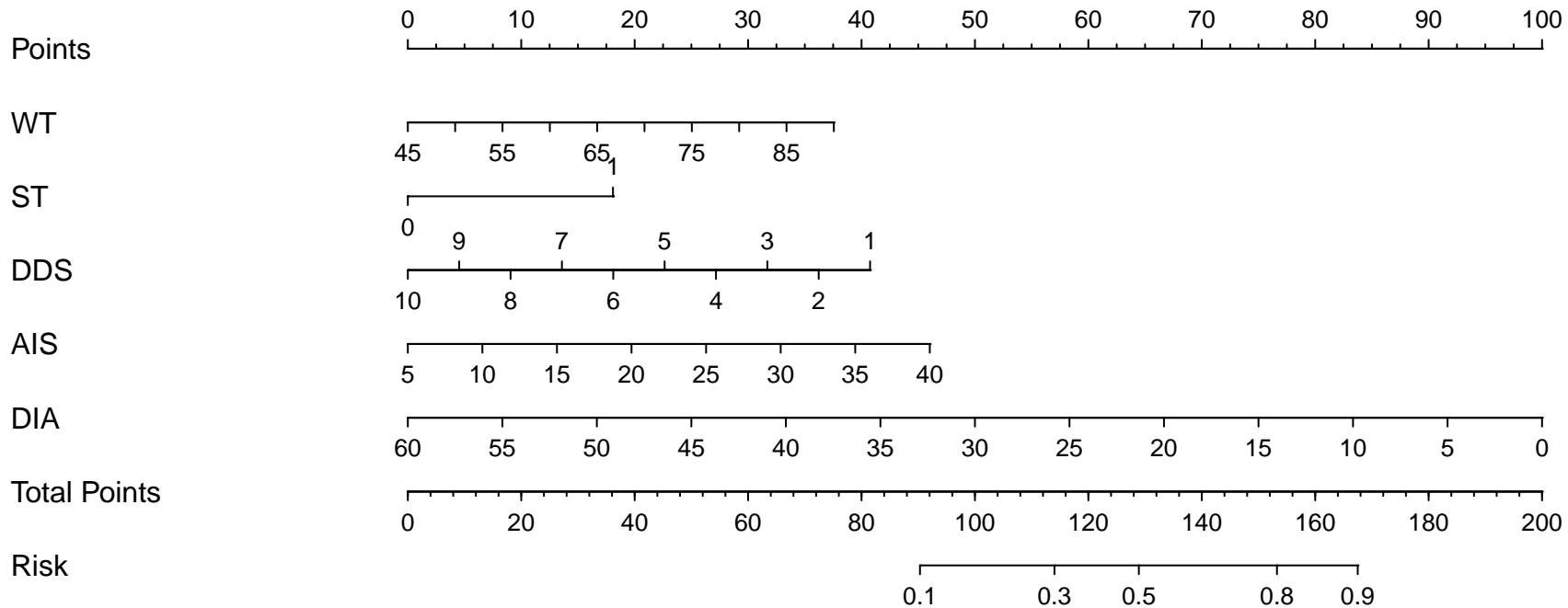

Supplement: Supplementary file 4 [file DataSheet4.pdf]

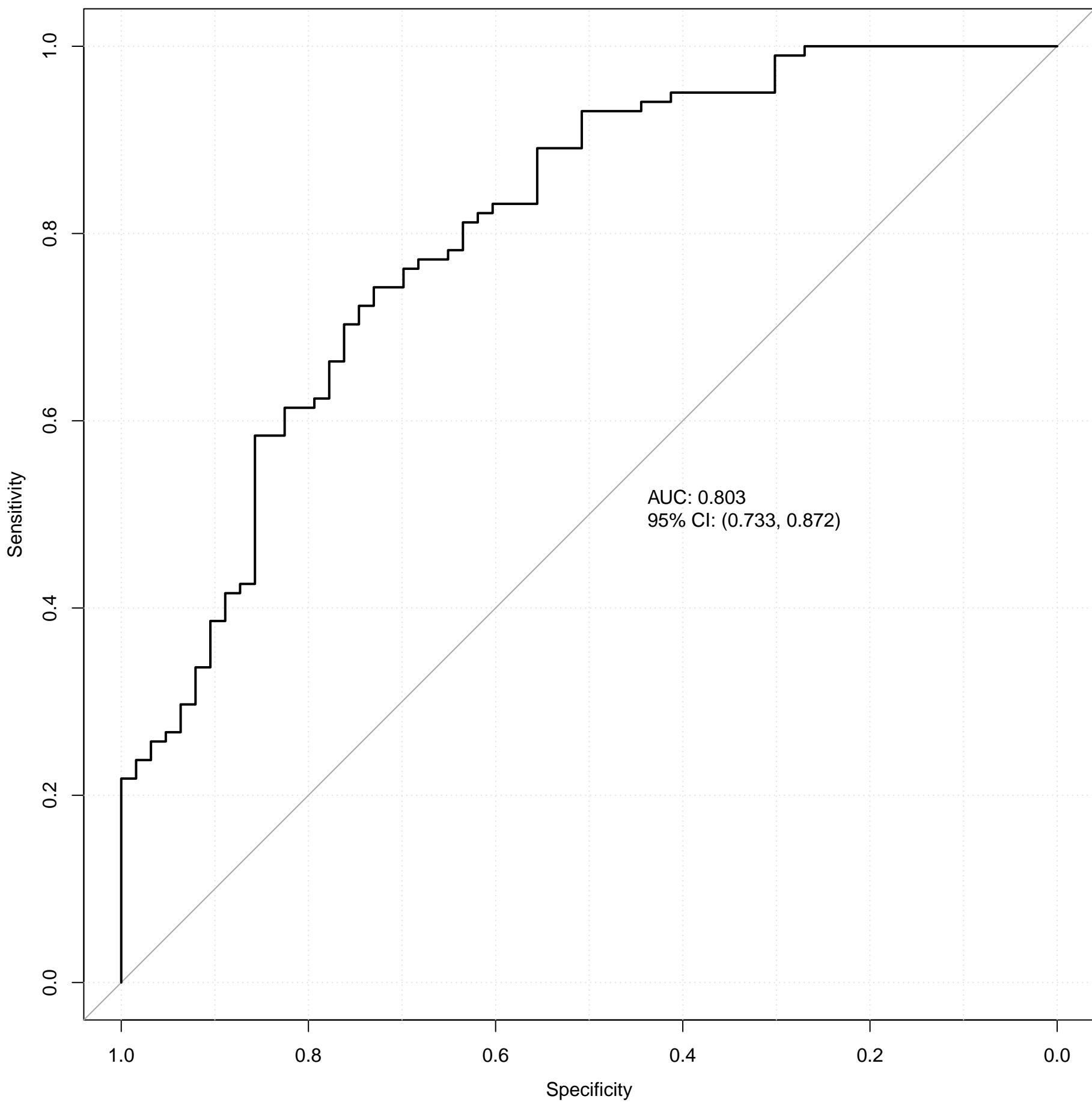

Supplement: Supplementary file 5 [file DataSheet5.pdf]

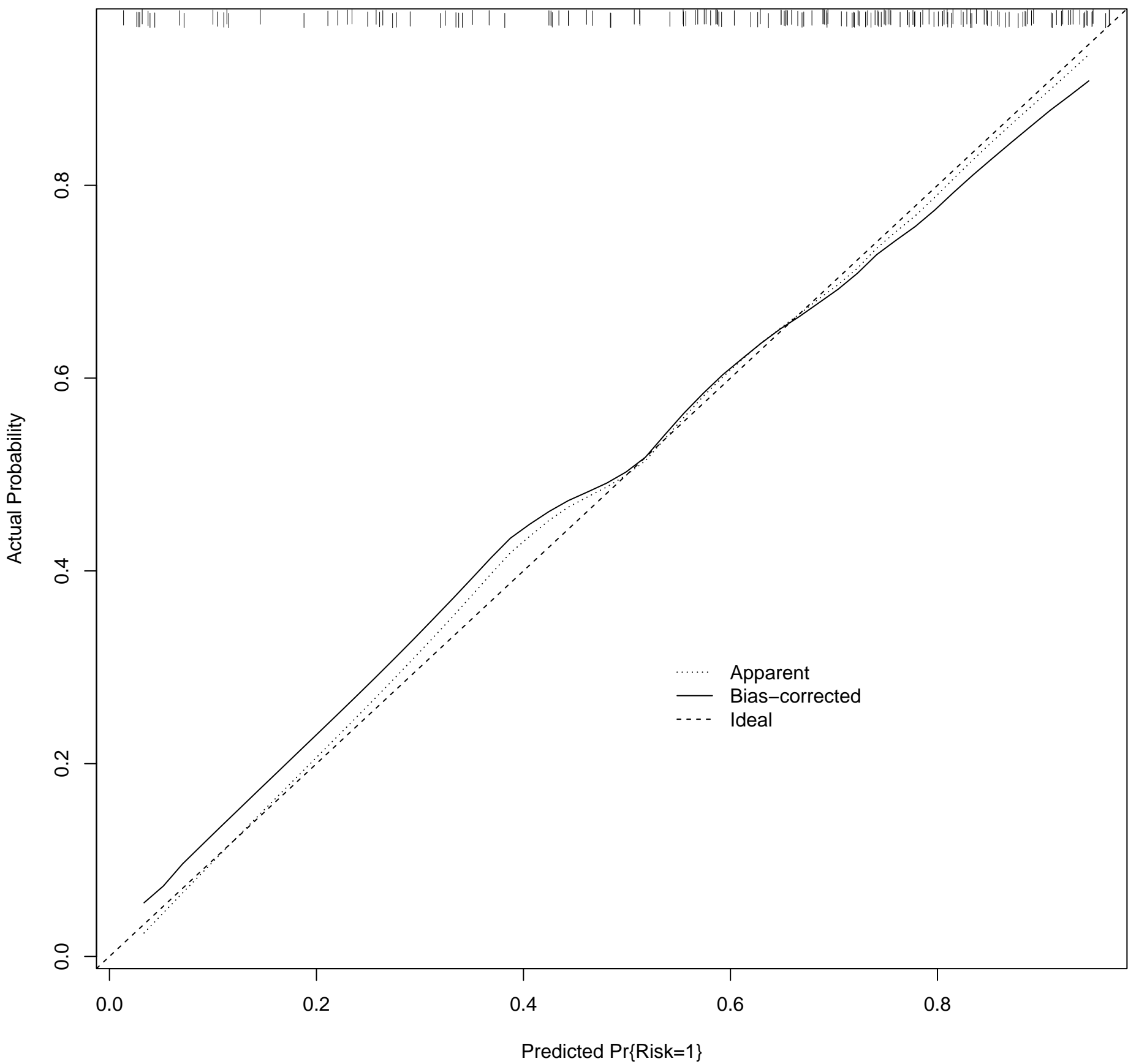

Supplement: Supplementary file 6 [file DataSheet6.pdf]

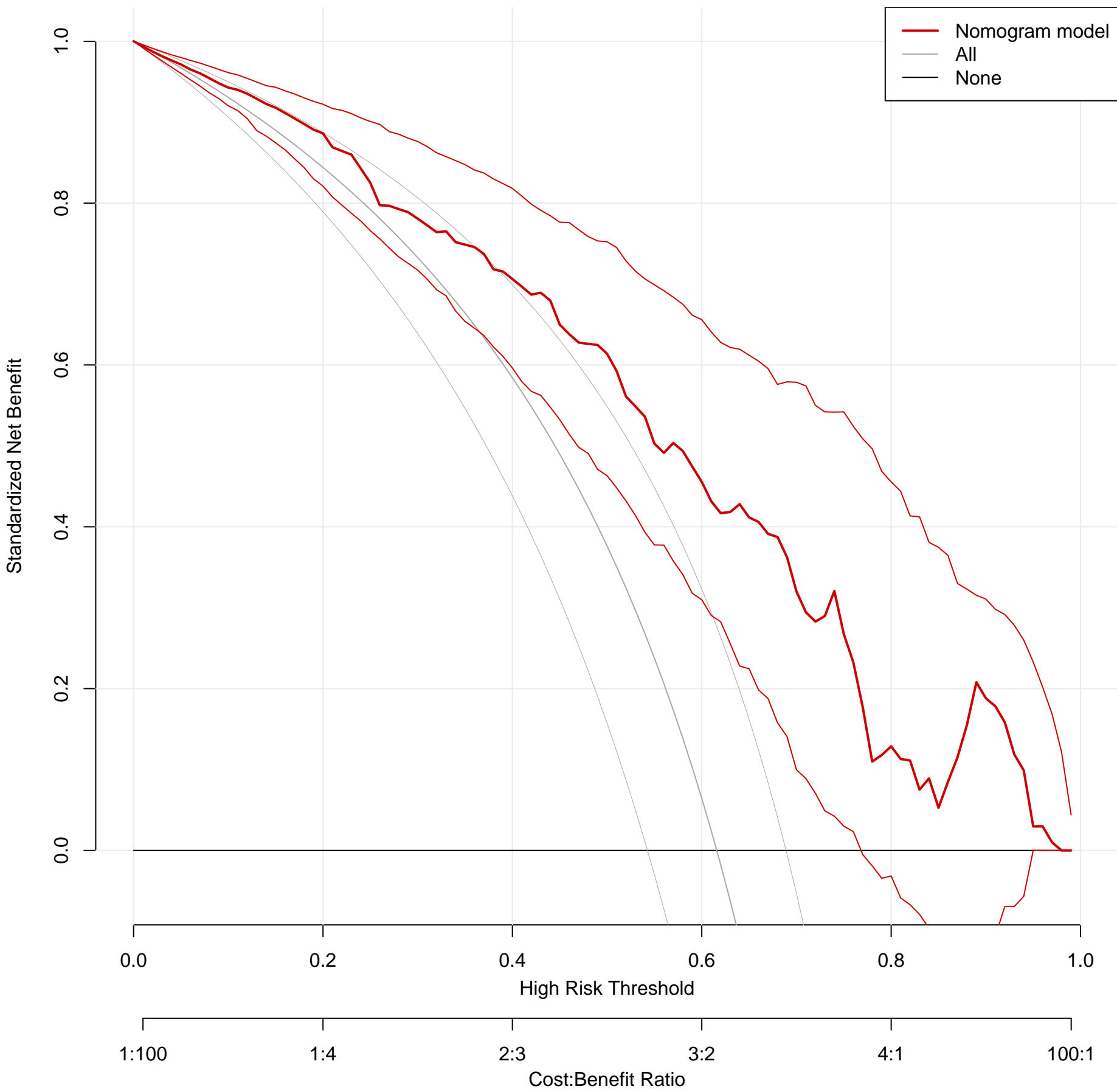

Supplement: Supplementary file 7 [file DataSheet7.pdf]
