# Supplementary material for: Development and validation of a predictive nomogram for high-risk thyroid nodules: a retrospective analysis of sedentary time, insomnia, and elevated weight
Source: Front Oncol. 2026 Apr 1;16:1698466. doi: 10.3389/fonc.2026.1698466 (PMC13080605; doi:10.3389/fonc.2026.1698466)
Supplement: Supplementary file 8 [file DataSheet8.pdf]

Table S1

AIS

0 to 7 points: Insomnia without clinical significance (normal sleep); 8 to 14 points: Subclinical insomnia (mild); 15 to 21 points: Clinical insomnia (moderate); 22 to 28 points: Clinical insomnia (severe)				
1 Describe the severity of your difficulty falling asleep over the past two weeks.				
None (0)	Slightly (1)	Moderately (2)	Severely (3)	Very severe (4)
2 Describe the severity of your difficulty in maintaining sleep over the past two weeks.				
None (0)	Slightly (1)	Moderately (2)	Severely (3)	Very severe (4)
3 Describe the severity of waking up too early in the past two weeks.				
None (0)	Slightly (1)	Moderately (2)	Severely (3)	Very severe (4)
4 How satisfied are you with your current sleep pattern?				
Very satisfied (0)	Satisfied (1)	Average (2)	Dissatisfied (3)	Very dissatisfied (4)
5 To what extent do you think your sleep problems interfere with your daily life				
None (0)	Slightly (1)	Quite (2)	Extremely (3)	Very extremely (4)
6 To what extent does your insomnia affect or impair your quality of life				

compared to others?				
None (0)	Slightly (1)	Quite (2)	Extremely (3)	Very extremely (4)
7 How worried or distressed are you about your current sleep problems?				
None (0)	Slightly (1)	Quite (2)	Extremely (3)	Very extremely (4)
