# Supplementary material for: Development and validation of a predictive nomogram for high-risk thyroid nodules: a retrospective analysis of sedentary time, insomnia, and elevated weight
Source: Front Oncol. 2026 Apr 1;16:1698466. doi: 10.3389/fonc.2026.1698466 (PMC13080605; doi:10.3389/fonc.2026.1698466)
Supplement: Supplementary file 9 [file DataSheet9.pdf]

Table S2

DASS-21

Evaluation degree: 0 — does not meet; 1 — sometimes meets; 2 — often meets; 3 — always meets.<br>The 21 test items are scored from 0 to 3, and the factors for 7 subscales are calculated and assessed.<br>Stress Scale: 1, 6, 8, 11, 12, 14, 18<br>Anxiety Scale: 2, 4, 7, 9, 15, 19, 20<br>Depression Scale: 3, 5, 10, 13, 16, 17, 21<br>Description:<br>A stress score of ≤ 14 is considered normal, 15-18 is mild, 19-25 is moderate, 26-33 is severe, and ≥ 34 is very severe.<br>An anxiety score of ≤ 7 is considered normal, 8-9 is mild, 10-14 is moderate, 15-19 is severe, and ≥ 20 is very severe.<br>A depression score of ≤ 9 is considered normal, 10-13 is mild, 14-20 is moderate, 21-27 is severe, and ≥ 28 is very severe.	
1	I find it hard to calm down.
2	I feel dry in my mouth.
3	I don't seem to feel any pleasure or comfort at all.
4	I feel like I'm having difficulty breathing, such as wheezing or shortness of breath.
5	I find it difficult to take the initiative to start working.
6	I often overreact to things.
7	I feel trembly (for example, my hands are shaking).

8	I feel like I've used up a lot of my energy.
9	I worry about situations that might cause me to panic or make a fool of myself.
10	I feel like I have nothing to look forward to soon.
11	I feel restless and uneasy.
12	I find it hard to relax.
13	I feel depressed and downcast.
14	I can't tolerate anything that prevents me from continuing to work.
15	I feel like I'm on the verge of a breakdown.
16	I can't generate enthusiasm for anything.
17	I feel like I'm not much of a person.
18	I find myself easily annoyed.
19	Even without obvious physical activity, I feel that my heart rhythm is irregular.
20	I feel afraid for no reason.
21	I feel that life is meaningless.
